# Virtual prenatal visits associated with high measures of patient experience and satisfaction among average-risk patients: a prospective cohort study

**DOI:** 10.1186/s12884-023-05421-y

**Published:** 2023-04-06

**Authors:** Bethany Bruno, Mary Beth Mercer, Sabahat Hizlan, Julian Peskin, Paul J. Ford, Ruth M. Farrell, Susannah L. Rose

**Affiliations:** 1 Department of Obstetrics & Gynecology, Medical University of South Carolina, Charleston, SC 29425 USA; 2 Cleveland Clinic Lerner College of Medicine of Case Western Reserve University, Cleveland, OH 44195 USA; 3 Office of Patient Experience, Cleveland Clinic, Cleveland, OH 44195 USA; 4 OB/GYN and Women’s Health Institute, Cleveland Clinic, Cleveland, OH 44195 USA; 5 Center for Bioethics, Cleveland Clinic, Cleveland, OH 44195 USA; 6 Genomic Medicine Institute, Cleveland Clinic, Cleveland, OH 44195 USA

**Keywords:** Prenatal care, Obstetrics, Patient experience, Healthcare access, Remote monitoring, Survey research, Telehealth, Telemedicine, Videoconferencing, Virtual visits

## Abstract

**Background:**

Virtual visits have the potential to decrease barriers to prenatal care stemming from transportation, work, and childcare concerns. However, data regarding patient experience and satisfaction with virtual visits remain limited in obstetrics. To address this gap, we explore average-risk pregnant women’s experiences with virtual visits and compare satisfaction with virtual vs. in-person visits as a secondary aim.

**Methods:**

In this IRB-approved, prospective cohort study, we surveyed pregnant women after their first virtual visit between October 7, 2019 and March 20, 2020. Using heterogeneous purposive sampling, we identified a subset of respondents with diverse experiences and opinions for interviews. For comparison, Consumer Assessment of Healthcare Providers and Systems (CAHPS) satisfaction data were collected after in-person visits during the study timeframe from a control cohort with the same prenatal providers. Logistic regression controlling for age, previous pregnancies, and prior live births compared satisfaction data between virtual and in-person visits. Other quantitative survey data were analyzed through descriptive statistics. Free text survey responses and interview data were analyzed using content analysis.

**Results:**

Ninety five percent (*n* = 165/174) of surveys and 90% (*n* = 18/20) of interviews were completed. Most participants were Caucasian, married, and of middle to high income. 69% (114/165) agreed that their virtual appointment was as good as in-person; only 13% (21/165) disagreed. Almost all (148/165, 90%) would make another virtual appointment. Qualitative data highlighted ease of access, comparable provider-patient communication, confidence in care quality, and positive remote monitoring experiences. Recognizing these advantages but also inherent limitations, interviews emphasized interspersing telemedicine with in-person prenatal encounters. CAHPS responses after in-person visits were available for 60 patients. Logistic regression revealed no significant difference in three measures of satisfaction (*p* = 0.16, 0.09, 0.13) between virtual and in-person visits.

**Conclusions:**

In an average-risk population, virtual prenatal visits provide a patient-centered alternative to traditional in-person encounters with high measures of patient experience and no significant difference in satisfaction. Obstetric providers should explore telemedicine to improve access – and, during the ongoing pandemic, to minimize exposures – using patients’ experiences for guidance. More research is needed regarding virtual visits’ medical quality, integration into prenatal schedules, and provision of equitable care for diverse populations.

**Supplementary Information:**

The online version contains supplementary material available at 10.1186/s12884-023-05421-y.

## Introduction

Standard prenatal care involves 12–16 outpatient visits [1]. However, the inconvenience of office visits creates an access barrier [2]. Per a recent survey, women desire ten office visits supplemented by interval contact [3]. Virtual visits using videoconferencing provide a unique opportunity to adapt to these preferences, continuing obstetric care with greater engagement and monitoring than a phone call or electronic message.

Little data are available about virtual prenatal visits prior to the pandemic [4–8]. To prevent exposures, many providers have since incorporated telehealth [9–14]. However, the limited literature focuses on satisfaction rather than the broader process indicator of patient experience- a more comprehensive and objective measure to guide quality improvement [15]. Furthermore, the few pre-pandemic studies exclusively utilized nursing virtual visits [4–8], decreasing continuity with the primary obstetric provider. Continuity improves outcomes [16–19] and pregnant patients prefer a single clinician [7, 20]. Continuity remained an issue during the pandemic given staffing limitations [21], and one study exclusively examined audio-only interactions [22].

Our healthcare system is ideally positioned to address this gap given the volume of virtual appointments conducted even before the pandemic. Twenty-five attending obstetricians and certified nurse midwives (CNMs) performed nearly 1000 virtual visits for 550-plus pregnant women in their faculty practices between 2017 and 2019. At the provider’s discretion regarding medical appropriateness, average-risk patients may opt to complete routine prenatal visit(s) through a secure platform and often receive a blood pressure cuff and/or Doppler. Blood pressure cuffs and fetal heart rate Dopplers are provided because of the recommendation for assessment of maternal blood pressure and the presence of fetal cardiac activity at each routine prenatal visit [1]. In this study, we elicit women’s perspectives and experiences with virtual prenatal visits in surveys and interviews and compare satisfaction after virtual vs. in-person visits as a secondary aim.

## Methods

We surveyed and interviewed average-risk pregnant women after their first virtual prenatal visit. Recognizing this as a minimal risk study, the Cleveland Clinic Institutional Review Board waived the need for documented informed consent in accordance with relevant ethical guidelines and regulation. Eligible patients were 18 years or older, fluent in English, possessed decision-making capacity, and had a virtual visit with an obstetrician or CNM between October 7, 2019 and March 20, 2020 as part of the providers’ standard of care for each patient. The Cleveland Clinic Office of Patient Experience provided internal funding for this study.

### Survey design

Quantitative and qualitative questions elucidated patients’ experiences using the virtual platform (Appendix S1). To assess face validity, we pilot tested the survey with 8 women who had recent virtual prenatal visits. In response to their feedback, the survey was shortened to remove redundancy and optimize phrasing. To evaluate perceptions of physicians’ empathy, the survey incorporated the Jefferson Scale of Patient Perceptions of Physician Empathy (JSPPPE) [23]. For consistency with other Likert scales, the instrument was modified from a 7-point to a 5-point scale. Altogether these questions assessed patient experience, a broader conception than satisfaction, which is commonly assessed via Consumer Assessment of Healthcare Providers and Systems (CAHPS) surveys. Nevertheless, to directly compare satisfaction after virtual vs. in-person visits, we incorporated key CAHPS questions. These are not routinely asked after virtual visits, requiring primary data collection. Demographics were collected for sample characterization.

### Recruitment

Researchers identified eligible participants via the electronic medical record (EMR). Notably, virtual visit volume drastically increased towards the study’s end given the pandemic. To prevent confounding due to provider inexperience with virtual visits, only patients of providers using telemedicine pre-pandemic were invited to participate. Patients received an email with links to survey materials within a day of their appointment. Non-responders were contacted by email, then phone, and mailed a paper copy as a final contact. Data were securely housed in REDCap [24, 25], an electronic data capture platform.

Using heterogeneous purposive sampling, we identified a subset of survey respondents with diverse experiences and opinions for interviews (Appendix S2). Those selected received an email invitation with a linked audio recording release and were contacted to schedule an interview. Two researchers conducted the interviews. Recruitment concluded upon reaching data saturation [26]. Respondents received $15 for survey completion plus $25 for interview participation.

We identified pregnancy complications and outcomes of study participants via their EMR. To directly compare CAHPS measures of satisfaction, we also obtained data from a control cohort with in-person visits during the same timeframe with the same providers. Our hospital system already collects CAHPS surveys for in-person prenatal appointments, so this information was readily available.

### Analysis

Quantitative survey and EMR data were analyzed through descriptive statistics. Logistic regression, controlling for age, previous pregnancies, and prior live births, compared CAHPS data between virtual and in-person visits. Covariates were selected based on availability within the hospital’s CAHPS database and focused on obstetric history since past experiences may affect expectations for and satisfaction with prenatal care.

Content analysis was used to analyze the open-ended interview transcripts and free text survey responses [27]. Two analysts employed an iterative process of data immersion and theme identification to create a coding framework that was independently applied to each transcript. Coder agreement was evaluated in SPSS [28] using the kappa statistic [29]. The analysts reconciled their use of codes, which resulted in substantial agreement (0.74). Code frequencies were computed. One analyst coded free text surveys responses, the second analyst reviewed them, and the two reconciled areas of disagreement before computing code frequencies.

## Results

### Patient sample

Of 174 eligible patients, 165 completed the survey (95%). Twenty respondents were invited for a semi-structured interview and 18 participated (90%). Demographics are shown in Table 1; participants were primarily Caucasian, married, and of middle to high income. Respondents had virtual visits with 23 unique providers (18 obstetricians, 5 certified nurse midwives) who contributed different volumes of patients spanning from one to 33 each (Figure S1). According to the EMR, 52 respondents (52/162, 32%) work in healthcare. Respondents were average-risk; twenty-two had a history of one prior pregnancy complication (Table S1).

**Table 1:** Participant demographics and behavioral characteristics (*n* = 165 unless otherwise noted)

	**N (%)**		**N (%)**
**Provider Type**		**Ethnicity**	
Obstetrician Midwife	138 (84) 27 (16)	Hispanic	10 (6)
**Age**		**Race**	
18–25 26–30 31–35 36–40 ≥40	17 (10) 43 (26) 66 (40) 34 (20) 5 (3)	White Black Asian Multiracial	147 (89) 10 (6) 4 (2) 4 (2)
**Relationship Status**		**Number of Children**	
Married	146 (88)	Zero One Two-plus	40 (24) 77 (47) 43 (26)
**Education**		**Employment**	
≤ 8^th^ Grade High School/GED College 1–3 years College Graduate Graduate/Professional Degree	1 (1) 13 (8) 27 (16) 52 (32) 72 (44)	Full-Time Part-Time Multiple Jobs Homemaker Unemployed	94 (57) 37 (22) 4 (2) 19 (12) 11 (7)
**Annual Household Income**		**Insurance**	
≤ $19,999 $20,000-$49,999 $50,000-$74,999 $75,000-$99,999 ≥$100,000	8 (5) 12 (7) 20 (12) 26 (16) 96 (59)	Private Medicaid None	144 (87) 19 (12) 2 (1)
**Accessible Technology**		**Primary Transportation**	
Smartphone Tablet Laptop Desktop	159 (96) 108 (65) 141 (85) 54 (33)	Personal Car Shared Car Family/Friend	157 (95) 7 (4) 1 (1)
**Commute to Provider’s Office (Miles)**		**Commute to Provider’s Office (Minutes)**	
≤5 6–10 11–15 > 15	41 (25) 56 (34) 33 (20) 35 (21)	≤10 11–20 21–30 > 30	26 (16) 79 (48) 39 (24) 21 (13)
**Prior Pregnancies**		**Previous Virtual Visits**	
Prior pregnancy Prior loss	130 (79) 52 (32)	Prenatal Care Other Healthcare Both	9 (5) 23 (14) 1 (1)
**Number of Virtual Prenatal Visits **(*n* = 161, excluding 4 who transferred care)		**Gestational Age at First Virtual Visit**	
1–2 3–4 5–6 7–8	66 (41) 69 (43) 23 (14) 3 (2)	≤11 6/7 12 0/7 – 19 6/7 20 0/7 – 27 6/7 28 0/7 – 35 6/7 36 0/7 – 40 0/7	5 (3) 48 (29) 72 (44) 35 (21) 4 (2)

### Decision for virtual visits

Among those interviewed, the provider initiated discussion of potential virtual visits. This typically took place in person (12, 67%) at an early visit (10, 56%), but sometimes occurred via phone (3, 17%) or the online patient portal (3, 17%). Table 2 reports interviewee’ initial reactions, concerns, and decision-making factors in pursuing virtual care. Most opted for virtual visits because of convenience (13, 72%). The three interviewees with virtual visits in March (17%) cited limiting potential exposures as central to their decision-making. Similarly, survey respondents (32/163, 20%) cited safety during the COVID-19 pandemic as the primary impetus for them to pursue virtual care.

**Table 2:** Interviews: patient reactions and decisions about virtual visits (*n* = 18)

Code	n (%)	Illustrative Quote(s)
**Initial Reaction**		
Excited	9 (50)	“I was like oh, that’s awesome!”
Surprised	9 (5)	“I was like, wait, that’s an option? I was shocked.” "I kind of questioned having a virtual visit living 10–15 min away… I think of telemedicine… in rural areas… [without] access to [doctors] close by."
Ambivalent	6 (33)	"It was fine. I was like ok, that’s an option, we can try it."
Relieved (avoid COVID)	2 (11)	“Due to [COVID-19], I felt relieved.”
**Initial Concerns**		
None		"I had no concerns because I really trust [my doctor]… If she [was] concern[ed], she would tell me… to come [in]."
Advance set-up	10 (56)	
Tech savvy	3 (17)	
Trust in provider	1 (6)	
Low risk pregnancy	1 (6)	
Previous healthy pregnancy	1 (6)	
Work in medicine	1 (6)	
Virtual visit limitations	6 (33)	"When a baby’s involved, you want to ensure [everything's okay]. You can’t do as much virtually as in person.” "There’s a lot of benefit to going into the office and having a doctor lay hands on you.”
Self vital reliability	4 (22)	“You’re trying to [take your] blood pressure [and the baby's] heartbeat at home [when] obviously your doctor knows more than you do."
**Decision-Making Factors**		
Convenience	13 (72)	"I’m a very laid-back person, so [it’s] a lot of traveling for a quick check-up." **“**I don’t want to leave the house, I’m too tired. Getting [my kids] in and out of the car… –…we can do this at home now.”
No travel	9 (50)	
For work	7 (39)	
For childcare	5 (28)	
Comfort of home	2 (11)	
Patient trust in and loyalty to provider/institution	7 (39)	“I trust them implicitly.” "I had a life-threatening ectopic pregnancy, and [my doctor] rescued me. So I had a lot of confidence in him.” “It was better to virtually see her than to see another doc.”
Patient comfort	4 (22)	“I felt comfortable with it.” “Two days before my virtual visit I had a hematologist appointment, so my vitals and everything were checked then… that also made me more comfortable.”
Provider comfort	3 (17)	“My doctor was confident and comfortable with it.” "If they’re giving me an option, it’s because they’re okay either way."
Limiting exposures	3 (17)	“[T]he more I c[an] do to protect my unborn baby and family I will do.” "[Until COVID] I did [in-person visits,] I guess just because we’re creatures of habit."
No expense	2 (11)	"I’m a single mom so it’s really hard to balance everything… I’m glad… you didn't [need] special insurance."

### Overall experience and satisfaction

Survey respondents reported excellent experiences. Over two-thirds (114, 69%) agreed that the virtual encounter was as good as an in-person visit while 13% (21) disagreed (Table 3). A quarter (42, 26%) agreed that their virtual encounter was better than in-person. Most would make another virtual appointment (148, 90%) and recommend virtual visits to other pregnant women (138, 84%).

**Table 3:** Surveys: Patient experience and satisfaction

**Patient Experience** (*n* = 165)					
	Strongly disagree	Disagree	Neutral	Agree	Strongly agree
My virtual visit was as good as an in-person visit with my prenatal care provider	2 (1%)	19 (12%)	30 (18%)	38 (23)	76 (46%)
My virtual visit was better than an in-person visit with my prenatal care provider	7 (4%)	46 (27%)	70 (42%)	15 (9%)	27 (16%)
If considered appropriate by my provider, I would make another virtual prenatal appointment	0 (0%)	6 (4%)	11 (7%)	51 (31%)	97 (59%)
I would recommend virtual visits to other pregnant women	0 (0%)	4 (2%)	23 (14%)	51 (31%)	87 (53%)
**CAHPS Measures of Patient Satisfaction**					
	Very poor	Poor	Fair	Good	Very good
Degree to which the provider cared for you as person (*n* = 164, *n* = 60)	0 (0%), 1 (2%)^a^	0 (0%), 0 (0%)	2 (1%), 1 (2%)	25 (15%), 3 (5%)	137 (84%), 55 (92%)
Likelihood of your recommending this provider to others (*n* = 164, *n* = 60)	0 (0%), 1 (2%)	0 (0%), 0 (0%)	2 (1%), 1 (2%)	24 (15%), 2 (3%)	138 (84%), 56 (93%)
Likelihood of your recommending our practice to others (*n* = 163, *n* = 60)	0 (0%), 0 (0%)	0 (0%), 0 (0%)	3 (2%), 1 (2%)	23 (14%), 4 (7%)	137 (84%), 55 (92%)

^a^The first number (percent) indicates CAHPS responses by survey respondents after virtual visits. The second number (percent) indicates CAHPS responses available after in-person visits; these data are routinely collected by the institution

Table 3 shows responses to CAHPS measures. CAHPS data were available for 60 patients after in-person visits. Logistic regression revealed no statistically significant difference in three CAHPS measures of patient satisfaction between virtual vs. in-person prenatal visits (“degree to which the provider cared for you as a person,” *p* = 0.16; “likelihood of your recommending this care provider to others,” *p* = 0.09; and “likelihood of your recommending our practice to others”, *p* = 0.13).

### Access and convenience

Most survey respondents (158, 96%) reported that their virtual visit saved time and made it easy to get care (142, 86%). In qualitative responses, two-thirds noted convenience (103/155, 66%) as what they liked best. Similarly, interviewed participants described virtual visits as more convenient (17, 94%) (Table 4).

**Table 4:** Interviews: comparison of virtual to In-Person Visits (*n* = 18)

Code	n(%)	Illustrative Quote(s)
**Convenience**		
Convenient	17 (94)	"It's way more convenient. I asked for another." "The appointments [without] ultrasounds [are] just check vitals, share any concerns or symptoms, and you’re done. To achieve [that] from… home is pretty nice."
No travel	13 (72)	
With respect to work	8 (44)	
With respect to childcare	7 (39)	
Comfort of home	4 (22)	
Little to no wait time	6 (33)	
Productive while wait	4 (22)	
Shorter appointment	6 (33)	“It feels quicker because you’re not being checked in… and doing the weight and everything with the nurse.”
Not convenient	3 (17)	“It was and wasn’t [convenient], only because it was early morning and I hadn’t taken my daughter to pre-school yet, so I… was distracted by her.”
Coordinating childcare	2 (11)	
Appointment timing	1 (6)	
Longer wait	1 (6)	
Tech challenges	1 (6)	
Less stressful	2 (11)	"Bringing two kids in with me is hard. With one in pre-school, scheduling around that is stressful."
**Communication**		
Comparable	17 (94)	"He was the same as always- considerate and caring."
Empathic (felt cared for)	17 (94)	"I felt just as checked in on and cared for."
Natural with established relationship	7 (39)	“I could gather her personality, empathy, all of that… [With our] established relationship, it was natural."
More personal with video	5 (28)	"[It's] more personal when you can see their faces. You can see emotion and gauge the conversation."
Provider not rushed	4 (22)	“She’s always patient; she didn’t seem rushed."
More focused	3 (17)	"When you’re on the virtual, it’s more one-on-one [with fewer] distractions. Less interruptions, like people coming in and out of the room."
Provider rushed	2 (11)	"In my first [in-person] visit, she was more calm and thorough. This one she [spoke] fast; it was quick.”
**Medical Care**		
Quick routine visit of comparable quality	15 (83)	"I'm being taken care of just as well as if I went into the office… I'm getting the same amount of care." “For routine-type visits, it’s the same.”
Questions answered	10 (56)	"He really took his time and answered [my] questions… it was a really good appointment."
Limitations in testing/exam		
No urinalysis	8 (44)	"I feel more comfortable doing in-person visits because… they take the weight, the urine sample, they are better at finding the [baby’s] heartbeat.”
Doppler/blood pressure not by medical professional	5 (28)	
No weight or different scale	4 (22)	
No fundal height	2 (11)	
Clear instructions	3 (17)	“He gives me the same info I’d [get] in the office.”
Same time with provider	3 (17)	“The time spent with the doctor is comparable.”
Sacrificed for convenience	2 (11)	"I felt like I [gave] up some things… to get the convenience of… home."

### Patient-provider relationship

Patients perceived high provider empathy on the modified JSPPPE, with half (86/162, 53%) giving their provider a perfect score of 25/25 and 90% (147), a score of 20-plus when collating Likert scale measures together (Table 5). In interviews, nearly all thought virtual communication was comparable to in-person (17, 94%) (Table 4). Interviewees cited their established relationship as important to successful online dialogue (7, 39%).

**Table 5:** Surveys: Modified Jefferson Scale of patient perceived physician empathy

	Strongly disagree	Disagree	Neutral	Agree	Strongly agree
The virtual prenatal provider could view things from my perspective (see things as I see them). (*n* = 163)	2 (1%)	0 (0%)	8 (5%)	38 (23%)	115 (71%)
The virtual prenatal provider asked about what is happening in my daily life. (*n* = 164)	0 (0%)	5 (3%)	16 (10%)	42 (26%)	101 (62%)
The virtual prenatal provider seemed concerned about me and my family. (*n* = 164)	0 (0%)	2 (1%)	12 (7.3%)	37 (23%)	113 (69%)
The virtual prenatal provider understood my emotions, feelings, and concerns. (*n* = 163)	0 (0%)	2 (1%)	5 (3%)	34 (21%)	122 (75%)
The virtual prenatal provider is an understanding doctor/midwife. (*n* = 164)	1 (1%)	0 (0%)	2 (1%)	27 (17%)	134 (82%)

### Medical quality

Most interviewees (15, 83%) described their virtual visit as comparable to an office visit given its routine nature with no need for in-person testing (Table 4). One noted, “If I had anything physical that I was worried about [and] wanted her to look at…then that might be an issue. But I didn’t.” An established relationship was important to confidence in care quality: “I wouldn’t feel as reassured by [a virtual visit with] somebody else… because they don’t know me and my health.” A minority felt they sacrificed quality for convenience (2, 11%). For example, "I didn’t really feel like I got medical care because [the doctor] just told me everything looked okay and I didn’t have questions…it didn’t feel the same as the doctor’s office." Still, over 99% of those surveyed (162/163) understood next steps in their prenatal care, 98% (162/164) had their questions answered, and 96% (157/163) endorsed enough time during their visit (157, 96%).

### Technology

Most survey respondents (138, 84%) connected on mobile devices. Nearly all were comfortable with the platform (151, 92%) and easily saw (130, 79%), heard (139, 84%), and spoke (146, 89%) with their provider.

Interviewees appreciated email and text appointment reminders (4, 22%), their position in the waiting room queue (4, 22%), and notification when the visit started (3, 17%). As one stated, “It tells you when your doctor’s coming…it’s almost better than an office visit [when] you don’t know [and] sometimes wait an hour."

Nevertheless, 50 survey respondents (30%) reported technical difficulties, such as problems connecting (13, 26%), disconnection (5, 10%), lagging (7, 14%), and poor audio (8, 16%) or video (5, 10%). These issues worsened with increased strain during the pandemic, at which time five (10%) respondents also reported that the system was down. For 16% of survey respondents (26/165) and half of those with problems (26/50, 52%), technical difficulties required a phone call or other alternative.

### Remote monitoring

One hundred one survey respondents (61%) received a Doppler and blood pressure cuff, 10 (6%) received a Doppler only, and 54 (33%) received neither. Patients not given these technologies already had them at home, transitioned to virtual care at the last minute, or had vitals recently checked at another appointment. Remote monitoring was used for all virtual visits in which patients had a home Doppler and blood pressure cuff, with these values entered into their EMR by the provider.

Among survey respondents who received equipment, three-quarters were taught to use it (cuff 71, 70%; Doppler 83, 76%), and most agreed it was easy to use (cuff 95, 94%; Doppler 94, 87%). Some patients had problems during their visit (cuff 11, 10%; Doppler 13, 12%) and felt frustrated (5, 3%), stressed (1, 1%), or anxious (3, 2%) when they had difficulty. But, there was comfort in being able to go in person: “the doctor told me ‘if [you] don’t find it, don’t stress, [you] can always come in.'” Table 6 shows facilitators and challenges noted in interviews.

**Table 6:** Interviews: Facilitators and challenges in remote monitoring (*n* = 18)

Code	n (%)	Illustrative Quote(s)
**Facilitators**		
Easy to use	8 (44)	“[It was] pretty easy and self-explanatory.”
Hands-on instructions	6 (33)	“Before my virtual appointment, actually two appointments before, they showed me at each one just to make sure I didn’t forget.” “He did… walk me through how to do the Doppler – to put the gel on, and where you would most likely find it.”
Verbal instructions	5 (28)	“[The doctor] gave me tips and tricks to be successful.”
Self or family works in medicine	4 (22)	“I’ve ultrasounded people’s bellies before. I do not do it regularly, nor have I done it in years… but I’m familiar with the technology.”
Instructions provided (written, verbal, video)	4 (22)	“There’s written instructions in the [blood pressure cuff] box, but it also [tells you what to do] out loud when you turn it on… There was instructions in the[Doppler] box as well.”
Answered questions	3 (17)	“By the time the virtual visit came around… I felt confident because I had had the opportunity to ask questions.”
Medical grade quality	2 (11)	“I had a blood pressure cuff that I didn’t fully trust [with my last pregnancy]… [with virtual visits], I got access to equipment that is reliable.”
**Challenges**		
Difficulty using	6 (33)	“The first time we did have to punt a bit…it was [some] trial and error.” “[My baby] was moving around… so it was hard for me to find [his heartbeat].”
Equipment problem	4 (22)	“The batteries didn’t work, so I had to pull batteries out of the remote.” “The blood pressure cuff was… a large, and it’s too big, so it doesn’t read [my blood pressure accurately].”
Not confident	3 (17)	“I’m less comfortable in my abilities to get the readings… correct.” “The hardest part was… is this my heartbeat or the baby’s.”
No in-office instruction	3 (17)	“I think that they basically assumed we knew how to use it because we’ve been going to so many appointments.” “I didn’t get any instructions… I don’t even think there were instructions in the bag.”
Allotted insufficient time before visit	2 (11)	“Because I was running late and trying to get ready for my appointment, being rushed made it… more challenging.”

### Virtual visit applications

When asked which encounters would be appropriate to conduct virtually, most interviewees described routine appointments not requiring in-person testing (13, 72%). Some described low-risk (7, 39%), second trimester (8, 44%) pregnancies as optimal. Both the patient (9, 50%) and provider (8, 44%) should be comfortable meeting virtually. For example, some first-time moms and their providers might be uncomfortable "because they don’t know what to look for…And it’s their first pregnancy so…you don’t know how their body reacts." Furthermore, "A lot of people with first babies love to go in." "With a virtual appointment you don’t get that thrill.” Nevertheless, it could “[benefit] a first-time mom because you have [many] questions and…feel silly…going in…when it’s…nothing to worry about.” Multiple interviewees noted that virtual and in-person visits should be interspersed (5, 28%). As one stated, “Is it a perfect replacement? No. Would I do it for every visit? No. But it’s a really nice option for some.”

Interviewees considered virtual visits potentially inappropriate in the first (6, 33%) and third trimester (5, 28%) and high-risk pregnancies (6, 33%). Concerns about the first trimester largely stemmed from patient comfort and the need to establish a relationship: “I like to make sure I like [the doctor] in person [first], because they’re with me for the next year or so.” In contrast, third trimester concerns centered on risk: “the closer to [the] due date…I think any convenience that virtual visits provide is not worth the risk.” Still, some thought that even high-risk individuals later in pregnancy should be allowed virtual visits “so long as they’re not endangered [by] not com[ing] in;” it may actually serve them best if they are on bed rest and “don’t feel like getting up.”

### Suggestions for improvement

Sixty percent (74/123) of survey respondents noted opportunities for improvement, such as better audiovisual connection (19, 26%), resolution of virtual platform technical difficulties (8, 11%), increased bandwidth during the pandemic (5, 7%), real-time customer service (3, 4%), and improved Doppler/blood pressure cuff instruction (8, 11%). Five interviewees (28%) requested more virtual visit availability.

Nevertheless, patients were largely pleased with their experience. Asked for final thoughts, one interviewee noted “virtual visits serve a very important purpose.” Another commented, “a lot of people don’t see a doctor because it’s inconvenient… anything… to make care convenient… is helpful.”

## Discussion

In this primarily average-risk, middle-class, Caucasian population, virtual prenatal visits provided a patient-centered alternative to traditional in-person encounters with high measures of patient experience and no statistically significant difference in satisfaction. Over a quarter of survey respondents described their virtual encounter as better than an in-person visit, almost 70% stated that it was as good as an in-person one, and nearly all would make another virtual appointment. Qualitative data highlighted accessibility, comparable provider-patient communication, confidence in medical quality, and positive experiences with remote monitoring. Recognizing these advantages, but also virtual visits’ inherent limitations, respondents preferred a prenatal care schedule interspersing telemedicine with traditional in-person encounters.

Our sample reflected other pre-pandemic studies wherein patients choosing virtual care were more likely Caucasian, married, and of middle-to high-income [6]. Like previous researchers, we found no difference in satisfaction after in-person and virtual encounters [4, 6]. Participants emphasized convenience and the majority perceived high provider empathy. Literature demonstrates the importance of prenatal care continuity [7, 19, 20]; in this vein, interviewees commented on the benefits of an established relationship for comfort in choosing virtual visits, improved virtual communication, and confidence in medical quality. Building on prior research suggesting that patients are interested in at-home fetal monitoring [3, 30], participants appreciated the ability to monitor their baby’s health.

Although these results expand upon the few pre-pandemic studies examining virtual visits, they may differ from some colleagues’ anecdotal experiences in the wake of COVID-19. Indeed, lower measures of patient experience and satisfaction during the pandemic would be understandable given that virtual visits were pursued out of necessity rather than choice, with limited resources and experience to support such a rapid transition. Nevertheless, our data resembles that from pandemic-era studies [21] examining more diverse populations’ experiences [22, 31], which have also found comparable patient satisfaction between virtual and in-person visits, distinct advantages and disadvantages to each, and a preference for a combined schedule.

Our study has several limitations. Most importantly, despite an excellent response, our sample was not diverse regarding race/ethnicity, partnering, and socioeconomic status. However, these demographics reflect our study site population. They also follow documented pre-pandemic trends in telemedicine usage [6], which parallel former reimbursement policies wherein virtual visits were only covered under private insurances’ bundled payments, not Medicaid’s fee-for-service model. Still, this significantly limits generalizability, particularly as virtual visits become more routine as a result of healthcare changes responsive to the pandemic. Furthermore, this sample included women electing for virtual visits. It does not reflect the experiences of those who declined to utilize telehealth and those unable to use telehealth because of poor digital access. This limits the ability to assess how social determinants of health serve as facilitators or barriers to virtual visits. Additionally, CAHPS data available after in-person prenatal visits was limited with 60 respondents. Although unsurprising given the trend in low response rates nationwide [32–34], this may reduce both our power to detect a difference in patient satisfaction and our ability to generalize to wider populations of patients. Nevertheless, this was not the study’s primary aim and our findings are consistent with the literature [4, 6], including surveys of more diverse populations [31].

More research is needed to ensure virtual prenatal appointments do not perpetuate disparities in healthcare access, quality, and outcomes. Our sample population was largely Caucasian with private insurance and ready access to technology. Virtual visits may be inaccessible for patients with fewer resources, and the digitally disadvantaged are more likely to be members of populations at higher baseline pregnancy risk due to socioeconomic and other demographic variables [35]. Thus, future investigations must assess quality and outcomes to confirm safety [36], with particular attention paid to patient population and the equitable provision of care.

Additional research is also needed to determine the optimal virtual vs.in-person visit schedule. Our average-risk respondents had anywhere from one to eight virtual visits interspersed at different timepoints over the course of pregnancy. Researchers have already begun studying different models integrating virtual visits into prenatal care [4, 13, 37, 38]. A recent systematic review by the Agency of Healthcare Research & Quality addressed the preferred visit schedule and the use of telemedicine for routine antenatal care, but unfortunately concluded that evidence remains relatively sparse [39]. In developing new approaches, researchers should continue to explore diverse patients’ experiences, and also providers’ experiences [21, 40], as provider support is crucial for successful uptake. Nevertheless, our data provide an important starting point to inform efforts to incorporate virtual visits into new intra- and post-pandemic models of prenatal care.

This study fills an important gap in our understanding of patients’ perceptions of virtual prenatal visits outside crisis standards of care implemented during the pandemic. Its extensive examination of patient experience through both surveys and interviews also distinguishes it from other pre- and intra-pandemic studies that have largely focused on the narrower concept of satisfaction. Additionally, our virtual prenatal care model uniquely maintains continuity with patients’ primary obstetric providers compared to other published approaches. As we transition to a post-COVID world with renewed focus on innovative prenatal schedules, these data uniquely inform how virtual visits may be integrated into routine care.

## Conclusions

In an average-risk population, virtual prenatal visits provide a convenient, patient-centered alternative to traditional in-person encounters with confidence in medical quality, positive remote monitoring experiences, and no difference in satisfaction. Contrary to concerns about potential negative effects on patient-provider relationships [41] and the ability to express empathy [42], our data suggest that providers can continue collaborative relationships and demonstrate empathy in digital settings just as effectively as in the office. Although COVID forced rapid implementation of telemedicine services to reduce infectious exposures, our findings provide evidence that virtual visits should continue in a post-pandemic future to reduce office visit burden while maintaining provider communication and fetal monitoring. Moving forward, patients and providers should engage in shared decision-making regarding interspersing telemedicine with traditional in-person encounters. Furthermore, minimizing technical problems should be a priority given the potential impact on patient experience, satisfaction, care quality, and future uptake of telemedicine.

At a time when only 75% of women receive adequate prenatal care [43] – and during the ongoing pandemic, in which virtual visits minimize potential exposures – obstetric providers should explore telemedicine applications to improve access, using patients’ experiences for guidance. More research is needed regarding virtual visits’ medical quality, integration into prenatal care schedules, and provision of equitable care for diverse patient populations.

## Supplementary Information

**Additional file 1:** **Appendix S1.** Patient Survey. **Appendix S2.** Interview Guide. **Figure S1.** Distribution of respondents among providers. **Table S1.** Pregnancy compilations and outcomes.

## Data Availability

The datasets are not publicly available due to the hospital policy and personal privacy, but are available from the corresponding author on reasonable request.

## Abbreviations

CAHPS: Consumer Assessment of Healthcare Providers and Systems
CNM: Certified nurse midwife
COVID: Coronavirus disease
EMR: Electronic medical record
JSPPPE: Jefferson Scale of Patient Perceptions of Physician Empathy
SPSS: Statistical Package for the Social Sciences
