# Supplementary material for: Virtual prenatal visits associated with high measures of patient experience and satisfaction among average-risk patients: a prospective cohort study
Source: BMC Pregnancy Childbirth. 2023 Apr 6;23:234. doi: 10.1186/s12884-023-05421-y (PMC10077310; doi:10.1186/s12884-023-05421-y)
Supplement: Supplementary file 1 — Additional file 1: Appendix S1. Patient Survey. Appendix S2. Interview Guide. Figure S1. Distribution of respondents among providers. Table S1. Pregnancy compilations and outcomes. [file 12884_2023_5421_MOESM1_ESM.docx]

**Section 1: YOUR VIRTUAL PRENATAL CARE VISIT**

1. Why did you choose to see your prenatal care provider for a virtual visit instead of in-person appointment? **______________________________________________________________**

**_________________________________________________________________________**

**_________________________________________________________________________**

1. **Please indicate how much you agree or disagree with each statement below by checking one answer for each item.**

|  | **Strongly Disagree** | **Disagree** | **Neutral** | **Agree** | **Strongly Agree** |
| --- | --- | --- | --- | --- | --- |
| The wait time to see the virtual prenatal care provider was reasonable. | 🞎 | 🞎 | 🞎 | 🞎 | 🞎 |
| The virtual prenatal care provider answered my questions about my pregnancy. | 🞎 | 🞎 | 🞎 | 🞎 | 🞎 |
| I understand what I need to do next for my prenatal care. | 🞎 | 🞎 | 🞎 | 🞎 | 🞎 |
| I had enough time with the prenatal care provider during my virtual visit. | 🞎 | 🞎 | 🞎 | 🞎 | 🞎 |
| My privacy was respected during my virtual prenatal care visit. | 🞎 | 🞎 | 🞎 | 🞎 | 🞎 |

1. **Please indicate how much you agree or disagree with each statement below by checking one answer for each item.**

|  | **Strongly Disagree** | **Disagree** | **Neutral** | **Agree** | **Strongly Agree** |
| --- | --- | --- | --- | --- | --- |
| The virtual prenatal care provider could view things from my perspective (see things as I see them). | 🞎 | 🞎 | 🞎 | 🞎 | 🞎 |
| The virtual prenatal care provider asked about what is happening in my daily life. | 🞎 | 🞎 | 🞎 | 🞎 | 🞎 |
| The virtual prenatal care provider seemed concerned about me and my family. | 🞎 | 🞎 | 🞎 | 🞎 | 🞎 |
| The virtual prenatal care provider understood my emotions, feelings, and concerns. | 🞎 | 🞎 | 🞎 | 🞎 | 🞎 |
| The virtual prenatal care provider is an understanding doctor/midwife. | 🞎 | 🞎 | 🞎 | 🞎 | 🞎 |

1. **Please rate the services you received from our practice by checking the response that best describes your experience.**

|  | **Very Poor** | **Poor** | **Fair** | **Good** | **Very Good** |
| --- | --- | --- | --- | --- | --- |
| Degree to which the provider cared for you as a person | 🞎 | 🞎 | 🞎 | 🞎 | 🞎 |
| Likelihood of your recommending this care provider to others | 🞎 | 🞎 | 🞎 | 🞎 | 🞎 |
| Likelihood of your recommending our practice to others | 🞎 | 🞎 | 🞎 | 🞎 | 🞎 |

**SECTION 2: TECHNOLOGY**

1. How did you connect for your virtual prenatal care visit?

🞎 iPhone, Android, or other smartphone

🞎 iPad or other tablet

🞎 Laptop

🞎 Desktop computer

1. Did you use the “invite a guest” option to add another person, such as a family member, to the virtual prenatal care visit?
   - Yes
   - No
   - I did not know that this was an option, but would have used it.
   - I did not know that this was an option, but would not have used it.
2. **Please indicate how much you agree or disagree with each statement below by checking one answer for each item.**

|  | **Strongly Disagree** | **Disagree** | **Neutral** | **Agree** | **Strongly Agree** |
| --- | --- | --- | --- | --- | --- |
| The online system was easy to use. | 🞎 | 🞎 | 🞎 | 🞎 | 🞎 |
| I was comfortable using the online system. | 🞎 | 🞎 | 🞎 | 🞎 | 🞎 |
| It was easy to see the prenatal care provider during my virtual visit. | 🞎 | 🞎 | 🞎 | 🞎 | 🞎 |
| It was easy to hear the prenatal care provider during my virtual visit. | 🞎 | 🞎 | 🞎 | 🞎 | 🞎 |
| It was easy to talk with the prenatal care provider during my virtual visit. | 🞎 | 🞎 | 🞎 | 🞎 | 🞎 |

1. Did you have any technical difficulties with the online system during your virtual prenatal care visit?
   - Yes
   - No

***IF YES***, please describe: **_____________________________________________________**

**_________________________________________________________________________**

**_________________________________________________________________________**

1. Did you receive a blood pressure cuff and/or Doppler to use for your virtual prenatal care visit?
   - Yes, I received BOTH a blood pressure cuff and Doppler. **🡪 Complete Parts A and B**
   - Yes, I received ONLY a blood pressure cuff. **🡪 Complete Part A only**
   - Yes, I received ONLY a Doppler. **🡪 Complete Part B only**
   - No, I did not received a blood pressure cuff or Doppler. **🡪 Proceed to Question 10**

**PART A**

**i. Please indicate how much you agree or disagree with each statement below by checking one answer for each item.**

|  | **Strongly Disagree** | **Disagree** | **Neutral** | **Agree** | **Strongly Agree** |
| --- | --- | --- | --- | --- | --- |
| I was taught how to use the blood pressure cuff. | 🞎 | 🞎 | 🞎 | 🞎 | 🞎 |
| The blood pressure cuff was easy to use. | 🞎 | 🞎 | 🞎 | 🞎 | 🞎 |

**ii.** Did you have any problems using the blood pressure cuff for your virtual prenatal care visit?

- - Yes
  - No

***IF YES***, please describe: **_____________________________________________________**

**_________________________________________________________________________**

**_________________________________________________________________________**

***IF YES***, how did these problems make you feel? **__________________________________**

**_________________________________________________________________________**

**_________________________________________________________________________**

**iii.** Have you used the blood pressure cuff outside of your virtual prenatal care visit?

- - Yes
  - No

***IF YES***, how often?

- - Less than once a week
  - 1 to 3 days per week
  - 4 to 6 days per week
  - Every day

**PART B**

**i. Please indicate how much you agree or disagree with each statement below by checking one answer for each item.**

|  | **Strongly Disagree** | **Disagree** | **Neutral** | **Agree** | **Strongly Agree** |
| --- | --- | --- | --- | --- | --- |
| I was taught how to use the Doppler (technology used to detect your baby’s heart rate). | 🞎 | 🞎 | 🞎 | 🞎 | 🞎 |
| The Doppler was easy to use. | 🞎 | 🞎 | 🞎 | 🞎 | 🞎 |

**ii.** Did you have any problems using the Doppler for your virtual prenatal care visit?

- - Yes
  - No

***IF YES***, please describe: **_____________________________________________________**

**_________________________________________________________________________**

**_________________________________________________________________________**

***IF YES***, how did these problems make you feel? **__________________________________**

**_________________________________________________________________________**

**_________________________________________________________________________**

**iii.** Have you used the Doppler provided outside of your virtual prenatal care visit?

- - Yes
  - No

***IF YES***, how often?

- - Less than once a week
  - 1 to 3 days per week
  - 4 to 6 days per week
  - Every day

**SECTION 3: OVERALL IMPRESSIONS**

1. **Please indicate how much you agree or disagree with each statement below by checking one answer for each item.**

|  | **Strongly Disagree** | **Disagree** | **Neutral** | **Agree** | **Strongly Agree** |
| --- | --- | --- | --- | --- | --- |
| The online system made it easy to get the prenatal care I needed. | 🞎 | 🞎 | 🞎 | 🞎 | 🞎 |
| My virtual prenatal care visit saved me time. | 🞎 | 🞎 | 🞎 | 🞎 | 🞎 |
| My virtual prenatal care visit was more convenient than an in-person visit. | 🞎 | 🞎 | 🞎 | 🞎 | 🞎 |
| For my prenatal care, my virtual visit was as good as an in-person visit with my prenatal care provider. | 🞎 | 🞎 | 🞎 | 🞎 | 🞎 |
| For my prenatal care, my virtual visit was better than an in-person visit with my prenatal care provider. | 🞎 | 🞎 | 🞎 | 🞎 | 🞎 |
| If considered appropriate by my provider, I would make another virtual prenatal care appointment. | 🞎 | 🞎 | 🞎 | 🞎 | 🞎 |
| I would make a virtual care appointment for other future non-pregnancy-related health concerns. | 🞎 | 🞎 | 🞎 | 🞎 | 🞎 |
| I would recommend virtual prenatal care visits to other pregnant women. | 🞎 | 🞎 | 🞎 | 🞎 | 🞎 |
| I would recommend virtual healthcare appointments to other patients. | 🞎 | 🞎 | 🞎 | 🞎 | 🞎 |

1. Please let us know what you liked best about your virtual prenatal care visit.

**________________________________________________________________________________________________________________________________________________________**

**____________________________________________________________________________**

1. Please let us know how we can improve our virtual prenatal care services.

**____________________________________________________________________________**

**____________________________________________________________________________**

**____________________________________________________________________________**

**Section 4: YOUR EXPERIENCE WITH IN-PERSON VISITS**

1. How far do you usually travel to get to your prenatal care provider’s office?

🞎 5 or less miles

🞎 6 to 10 miles

🞎 11 to 15 miles

🞎 16 to 20 miles

🞎 More than 20 miles

1. How long does it usually take you to get to your prenatal care provider’s office?

🞎 10 or less minutes

🞎 11 to 15 minutes

🞎 16 to 20 minutes

🞎 21 to 25 minutes

🞎 26 to 30 minutes

🞎 More than 30 minutes

1. From the time of your scheduled appointment to when you see your prenatal care provider, how long do you usually wait in the office?

🞎 10 or less minutes

🞎 11 to 20 minutes

🞎 21 to 30 minutes

🞎 31 to 40 minutes

🞎 41 to 50 minutes

🞎 51 to 60 minutes

🞎 More than 1 hour

1. How long do you usually meet with your prenatal care provider in the office?

🞎 5 or less minutes

🞎 6 to 10 minutes

🞎 11 to 15 minutes

🞎 16 to 20 minutes

🞎 More than 20 minutes

**Section 5: OTHER INFORMATION ABOUT YOU**

1. What is your age?

🞎 18-20

🞎 21-25

🞎 26-30

🞎 31-35

🞎 36-40

🞎 Over 40

1. Are you Hispanic or Latina?

🞎 Yes

🞎 No

1. How would you describe your race? **Please check all that apply.**

🞎 American Indian or Alaska Native

🞎 Asian

🞎 Black or African American

🞎 Native Hawaiian or Other Pacific Islander

🞎 White

1. What is your relationship status?

🞎 Single

🞎 In a relationship

🞎 Married

1. Have you been pregnant before?

🞎 Yes

🞎 No

***IF YES***, have you had a prior pregnancy loss (also known as a miscarriage)?

🞎 Yes

🞎 No

1. Do you have children?

🞎 Yes

🞎 No

***IF YES***, how many children do you have?

🞎 One child

🞎 Two children

🞎 Three children

🞎 Four or more children

***IF YES***, how old are your children? **Please check all that apply.**

🞎 Less than 1 year old

🞎 1 to 3 years old

🞎 4 to 6 years old

🞎 7 to 9 years old

🞎 10 to 12 years old

🞎 13 to 15 years old

🞎 16 to 18 years old

🞎 Older than 18 years old

1. What is the highest grade or school year that you have completed?

🞎 Under Grade 8 (No high school)

🞎 Grades 9-11 (Some high school)

🞎 Grade 12 or GED (High school graduate)

🞎 College 1-3 years (Some college, technical school, or associate’s degree)

🞎 College 4 years or more (College graduate)

🞎 Graduate or professional degree

1. What is your employment status? Select all that apply.

🞎 Unemployed and not seeking work

🞎 Unemployed and seeking work

🞎 Work part-time (<40 hours per week)

🞎 Work full-time (40+ hours per week)

🞎 Work multiple jobs

🞎 Homemaker

1. What is your health insurance plan?

🞎 Medicaid

🞎 Medicaid plus supplemental private insurance

🞎 Medicare

🞎 Medicare plus supplemental private insurance

🞎 Private insurance

🞎 I do not have health insurance

1. What is your household’s current annual income?

🞎 $19,999 or less per year

🞎 $20,000 to $49,999 per year

🞎 $50,000 to $74,999 per years

🞎 $75,000 to $99,999 per year

🞎 $100,000 or more per year

1. What forms of technology do you own or otherwise have access to? **Please check all that apply.**

🞎 iPhone, Android, or other smartphone

🞎 iPad or other tablet

🞎 Laptop

🞎 Desktop computer

🞎 None of the above

1. What is your primary method of transportation?

🞎 I drive my own car

🞎 I drive a car I share with another person

🞎 A family member or friend drives me

🞎 I take a taxi or use a rideshare service such as Uber or Lyft

🞎 I take public transportation

🞎 I walk

🞎 Other: ________________________

1. Had you had a virtual visit before your most recent prenatal care appointment (at Cleveland Clinic or another healthcare system)?

🞎 No, this was my first virtual visit.

🞎 Yes, I had virtual prenatal care visits with a prior pregnancy.

🞎 Yes, I have had virtual visits for other aspects of my healthcare.

🞎 Yes, I have had BOTH virtual prenatal care visit(s) with a prior pregnancy and virtual visit(s) for other aspects of my healthcare.

**Thank you for completing this survey!**

You will receive a $15 Amazon gift card approximately one week after we receive your completed survey to thank you for your time. The electronic gift card will be sent via email to the address used for your virtual visit.

**Patient Experience with Virtual Prenatal Care Visits**

Patient Interview Guide

**Recording Interview** (check one and proceed accordingly**)**

🞎 I received your signed Release Form to record the phone interview. I will turn on the recorder

now.

🞎 I did not receive a signed Release Form to record the phone interview. However, we can proceed with the phone interview without recording it – I will take notes instead.

**Interview Questions**

1. How did you first hear about virtual prenatal care visits?
   1. What was your initial reaction?
   2. What concerns did you have, if any?
   3. What ultimately led to your decision to have a virtual prenatal care visit?
2. How did your virtual prenatal care visit compare to an in-person visit with your prenatal care provider?
   1. With regard to convenience?
   2. With regard to the interaction with your prenatal care provider?
   3. With regard to medical care quality?
3. Tell us about your experience using the home blood pressure cuff and doppler (technology used to detect your baby’s heart rate).
4. Would you make another virtual prenatal care appointment? Why or why not?
5. Based on your experience, when do you think a virtual prenatal care visit is most appropriate?
   1. To whom should we offer virtual prenatal care visits?
   2. For which prenatal care appointments should we offer a virtual visit?
6. Based on your experience, what could we do to improve our virtual prenatal care services?
7. Would you be interested in virtual visits for other aspects of your medical care?
   1. If no, why?
   2. If yes, why and in which circumstances?

Respondents had virtual visits with 23 unique providers (18 obstetricians, 5 certified nurse midwives) who contributed different volumes of patients. The x-axis delineates with how many respondents a provider had virtual visits. The y-axis delineates how many obstetricians (OB) or certified nurse midwives (CNM) had virtual visits with that number of respondents. For example, six obstetricians had a virtual visit with only one respondent, whereas one obstetrician had virtual visits with 33 different respondents. Thus, while many providers had virtual visits with only a few respondents, some providers saw many more patients virtually.

|  | **N (%)** |
| --- | --- |
| **Advanced Maternal Age** | 57 (35) |
| **Pre-Pregnancy Body Mass Index**  Normal (18.5 – <25)  Overweight (25 – <30)  Obese (30 – <40)  Severely Obese (>40) | 87 (53)  41 (25)  30 (18)  7 (4) |
| **Substance Use**  Current Tobacco Use  Former Tobacco Use  Current Illicit Drug Use  Former Illicit Drug Use | 2 (1)  21 (13)  1 (1)  2 (1) |
| **Previous Birth Outcomes**  Prior Preterm Delivery  Prior Cesarean Section | 11 (7)  31 (19) |
| **Previous Pregnancy Complications**  History of Gestational Diabetes  History of Gestational Hypertension  History of Preeclampsia  History of HELLP Syndrome  History of Intrauterine Growth Restriction | 3 (2)  4 (2)  6 (4)  1 (1)  3 (2) |
| **Pre-Existing Conditions**  Diabetes Mellitus  Chronic Hypertension | 0 (0)  5 (3) |
| **Birth Outcomes** (n=161; 4 transferred care)  Neonatal ICU Admission  Low Birth Weight (<2500 grams)  Preterm Delivery (<37 weeks)  Early Preterm (<34 weeks)  Late Preterm (34 – 36 6/7 weeks)  Cesarean Section  Operative Vaginal Delivery  Postpartum Hemorrhage | 6 (4)  8 (5)  0 (0)  6 (4)  50 (31)  5 (3)  18 (11) |
| **Pregnancy Complications** (n=161; 4 transferred care)  Gestational Diabetes  Gestational Hypertension  Preeclampsia  HELLP Syndrome  Intrauterine Growth Restriction | 13 (8)  7 (4)  7 (4)  1 (1)  4 (2) |
